# Simultaneous tDCS-fMRI reveals limited and inconsistent changes in functional connectivity: Insights from a temporal dynamics study

**DOI:** 10.1162/IMAG.a.1109

**Published:** 2026-04-02

**Authors:** Debby C.W. Klooster, Guo-Rong Wu, Sara de Witte, Koen Kaalberg, Boaz Kalkhoven, Rob M.C. Mestrom, Chris Baeken

**Affiliations:** Eindhoven University of Technology, Department of Electrical Engineering, Electromagnetics for Care and Cure, Flux Building, Eindhoven, The Netherlands; Ghent Experimental Psychiatry Laboratory, Department of Head and Skin, Ghent University, Ghent, Belgium; Key Laboratory of Cognition and Personality, Faculty of Psychology, Southwest University, Chongqing, China; Neuroprotection and Neuromodulation Research Group (NEUR), Center for Neurosciences (C4N), Vrije Universiteit Brussel, Brussel, Belgium; Department of Neurology and Bru-BRAIN, Universitair Ziekenhuis Brussel, Brussel, Belgium; Department of Psychiatry, University Hospital Brussels, Brussels, Belgium

**Keywords:** transcranial direct current stimulation, resting-state functional MRI, functional connectivity, electric field simulations

## Abstract

Transcranial direct current stimulation (tDCS) is a non-invasive form of neuromodulation. Previous work has shown that tDCS affects functional connectivity, typically assessed by comparing resting-state functional MRI (rs-fMRI) data collected before and after the intervention. This study focuses on the temporal dynamics of functional connectivity during tDCS. Additionally, electric field simulations are incorporated in functional connectivity analyses to gain more insights into the mechanism of action. Forty-seven healthy female volunteers were enrolled in a randomized, sham-controlled, cross-over design in which sham and active tDCS were administered to the left dorsolateral prefrontal cortex for 20 min at 1.5 mA. Functional connectivity analyses were performed on rs-fMRI data collected before, during, and after tDCS, using three seed regions in the brain: one under the anode, one under the cathode, and one at the brain region where the individual tDCS-induced electric field strength was highest. The rs-fMRI data collected during stimulation were divided into three time windows to obtain temporal information on functional connectivity during stimulation. Functional connectivity was assessed at the whole-brain level using seed-to-voxel analyses as well as within predefined resting-state networks. TDCS did not consistently change functional connectivity over time. On the whole-brain level, active tDCS did not affect functional connectivity during stimulation. After active stimulation, only the functional connectivity between the cathode and the postcentral gyrus was increased. At the network level, changes in functional connectivity were observed following both sham and active tDCS, indicating that these effects could not be specifically attributed to active stimulation. Future research should further investigate the relationship between tDCS-induced effects on functional connectivity and their potential links to clinical responses.

## 1 Introduction

Transcranial direct current stimulation (tDCS) is a non-invasive neuromodulatory intervention that has gained traction in the management of neuropsychiatric disorders (Brunoni et al., 2012; Fregni et al., 2021; Lefaucheur et al., 2017). It operates by applying low-amplitude electrical currents through scalp electrodes, generating weak electric fields that do not directly induce action potentials (Klooster et al., 2016; Woods et al., 2016). Instead, tDCS alters neuronal excitability by modulating resting membrane potentials, thereby increasing the propensity for depolarization or hyperpolarization (Klooster et al., 2016). While anodal and cathodal stimulation were once thought to respectively enhance and inhibit cortical excitability, recent findings suggest these effects are contingent on several variables. One important factor is stimulation duration. Despite 20-minute sessions being the standard in clinical trials, the optimal stimulation duration for maximizing and sustaining therapeutic effects remains unresolved.

Over the past two decades, tDCS has increasingly been combined with functional magnetic resonance imaging (fMRI) for both mechanistic investigations and brain mapping purposes, including concurrent acquisitions. Integrating tDCS with resting-state fMRI (rs-fMRI) enables direct observation of changes in neural activity and connectivity over time (Esmaeilpour et al., 2020). Systematic reviews have documented over a hundred tDCS–fMRI studies, and a consensus-based checklist—the ContES (Concurrent transcranial electric stimulation-fMRI) checklist—now provides standardized guidelines for reporting and methodology in concurrent tES–fMRI research (Ekhtiari et al., 2022). The safety and compatibility of MRI-compatible tDCS equipment have been well established, and broader tES safety guidelines confirm that combining tES with neuroimaging introduces no additional risk when standard procedures are followed (Antal et al., 2017).

It is now possible to quantify the effects of tDCS on brain function by examining whole-brain functional connectivity patterns derived from rs-fMRI data. This approach is particularly well-suited for tDCS, as its effects are not limited to the targeted stimulation site but extend across distributed brain networks. Researchers have increasingly investigated these widespread effects using a sequential design, comparing functional connectivity before and after tDCS administration (see Supplementary Material A for an overview of the literature) (Keeser et al., 2011; Park et al., 2013; Peña-Gómez et al., 2012). Collectively, these findings confirm that tDCS can modulate functional connectivity beyond the stimulation site and influence global brain function.

Despite growing evidence, the precise neural mechanisms engaged during a tDCS session remain incompletely understood. In particular, it is still unclear which brain regions are most affected by tDCS and cause the effects on functional connectivity. Also, it is not known whether—and how—the effects of tDCS on functional connectivity evolve or accumulate over the course of stimulation. To address the latter point, recent studies have begun acquiring rs-fMRI data concurrently with tDCS (see Supplementary Material A) (Bouchard et al., 2023; Leaver et al., 2022; Mondino et al., 2020; Tu et al., 2021). While these studies collected rs-fMRI data during stimulation, most treated connectivity as a stationary measure—calculating metrics over the entire time series. Notably, only Mondino et al. (2020) divided the rs-fMRI acquisition into two time windows, allowing for an initial exploration of potential temporal dynamics during tDCS. This study showed that tDCS induced changes in functional connectivity between the left DLPFC and bilateral parietal regions already within the first 15 minutes of stimulation, that lasted in the second 15 minutes and after stimulation. Tu et al. (2021) also explicitly focused on the time-varying effects of tDCS on functional connectivity by investigating co-activation patterns (CAPs) derived from rs-fMRI data, using the regions under the stimulation electrodes as seed regions. Whereas seed-to-voxel connectivity focuses on correlations between time-series, CAPs focus more on spatial activation patterns that reoccur over time. The study found that tDCS significantly altered the occurrence rates of these CAPs indicating temporal effects of tDCS.

In the current study, we seek to extend previous findings by integrating tDCS-induced electric field simulations into functional connectivity analyses. Using the simulated electric field distributions, we defined seed regions for connectivity analyses. Comparable methodology has been employed in studies investigating the effects of transcranial magnetic stimulation (TMS) (Berger et al., 2024; Opitz et al., 2016). We hypothesize that the effects of tDCS on functional connectivity primarily originate from regions experiencing the strongest electric fields. In addition, we examine the temporal dynamics of these effects by applying a sliding-window approach to assess connectivity across successive time windows during stimulation. We predict that, compared to sham stimulation, active tDCS will modulate connectivity within distributed brain networks, with these effects progressively increasing over the course of the stimulation.

## 2 Methods

### 2.1 Inclusion criteria

For this study, we included 47 healthy right-handed young adult females, all within a close age range (mean age = 22.77 years old, standard deviation = 2.53 years) to eliminate gender effects and reduce age variability. The study was approved by the ethics committee of the university hospital of Ghent (UZGent). Each participant gave written informed consent.

### 2.2 Study design

In this double-blind, randomized, sham-controlled, cross-over design, active and sham tDCS (NeuroConn, DC-STIMULATOR MR) were applied during two study visits separated by at least 1 week. At the start of the study visit, anatomical T1-weighted MRI data (Siemens TrioTim 3T, Siemens, Erlangen, Germany, with a 32-channel SENSE head coil) were acquired (TR = 2250 ms, TE = 4.18s, FA = 9°, FOV = 256 x 256 mm, voxel size = 1 mm^3^, 176 slices). This scan was uploaded to the Brainsight neuronavigation system (BrainSight^TM^, Rogue Research, Inc) and guided electrode placement. The anodal tDCS electrode (5 x 5 cm) was placed over the left DLPFC; more specifically over the midprefrontal part of the left DLPFC. The cathodal electrode (5 x 5 cm) was placed over the contralateral orbitofrontal cortex (OFC), approximately 1 cm above the eyebrow. In the broader context of interventions targeting stress and depression, this electrode montage was chosen to align with standard tDCS protocols shown to enhance emotion regulation in depressed patients. This configuration was preferred over the bifrontal (left DLPFC–right DLPFC) setup, whose efficacy remains insufficiently supported by current evidence (Lefaucheur et al., 2017).

**Fig. 1.**
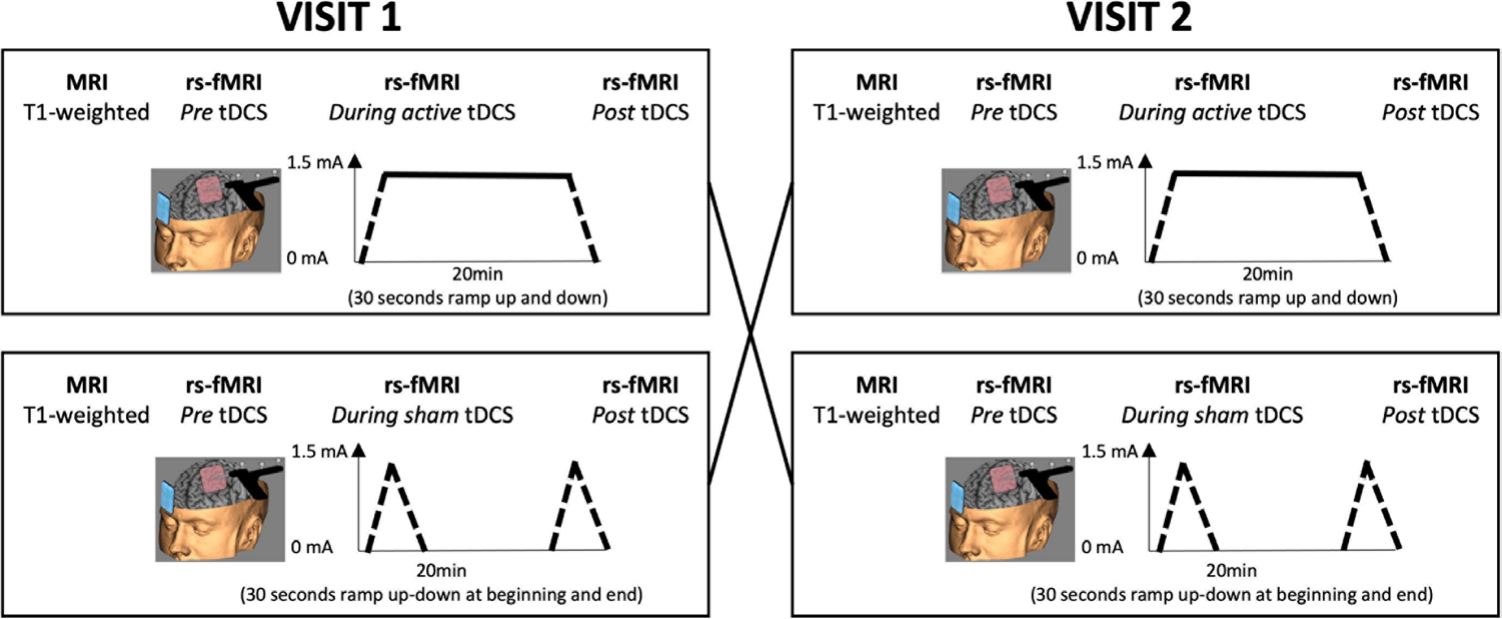
Schematic overview of the study design. Subjects were invited for two study visits in which active or sham tDCS was applied to the left DLPFC in randomized order. Rs-fMRI data were collected before, during, and after tDCS.

During both study visits, rs-fMRI data were collected before, during, and after tDCS (TR = 2500 ms, TE = 35 ms, FA = 80°, FOV = 224 × 224 mm; resolution = 3.5 × 3.5 × 3.0 mm; number of volumes = 170 for the pre- and post-scans and 530 during tDCS). Subjects were instructed to remain awake with their eyes closed. Active tDCS consisted of stimulation at 1.5 mA for 21 minutes, including a 30 second ramp-up and ramp-down period. For sham stimulation, the intensity was reduced immediately after the initial ramp-up period. This ramp-up and ramp-down pattern was repeated after 20 minutes. A schematic overview of the study design is shown in Figure 1.

### 2.3 Data analysis

#### 2.3.1 Preprocessing

Rs-fMRI data collected before, during, and after tDCS from both study visits were preprocessed using Matlab 2021a (The Mathworks Inc., Natrick, MA, US) and SPM12 (Wellcome Trust Centre for Neuroimaging, London, UK) according to the following standard steps. After realignment, correction for slice timing was applied and the data were smoothed with a 4 mm FWHM kernel. Confounding effects from motion (results of the realignment) and signals from the whole brain (global signal), white matter, and cerebrospinal fluid were regressed out. A high-pass filter with cut-off of 128 seconds was used. Finally, the data were normalized to MNI space.

#### 2.3.2 Definition of seed regions

For each subject, three circular seed regions with a radius of 1 cm were defined for functional connectivity analyses: one under the anode, one under the cathode, and one where the tDCS-induced electric field strength was strongest. Information from the subject-specific tDCS-induced electric fields was used either to derive weighting factors for the time series (all seeds) or to find the location of the seed (E_max_-seed). Electric field simulations were performed using Simnibs 3.2 (Thielscher et al., 2015). Individual head models were derived from the T1-weighted MRI data from the first study visit (Nielsen et al., 2018). The T1-weighted MRI data were segmented into different tissue types, and a head mesh was generated with different conductivity values assigned to the different tissue types. Using the default values in Simnibs*,* this means that the brain is segmented into ten different tissue types: white matter (0.126 S/m), gray matter (0.275 S/m), cerebrospinal fluid (1.654 S/m), bone (0.010 S/m), scalp (0.465 S/m), eye balls (0.500 S/m), compact bone (0.008 S/m), spongy bone (0.025 S/m), blood (0.600 S/m), and muscle (0.160 S/m). Together with information on the tDCS electrode positions, which was stored in the neuronavigation system, subject-specific tDCS-induced electric field distributions were derived.

Information from the individual electrode positions was used to derive circular seed regions under the anode and the cathode. These circular regions were masked with a gray matter mask. The regressors representing the anode and cathode were defined as the weighted average of the time-series of the voxels within the seed regions, with the normalized electric field strength as the weighting factor (Reg_DLPFC_ and Reg_OFC_).

In addition, for every subject a circular seed region was placed around the position where the tDCS-induced electric field strength was highest within the gray matter. To compensate for potential outliers, the maximum was defined as the 99.5% value of the electric field strengths in the region between the electrodes. The region between the electrodes was defined as the bounding box between x,y, and z coordinates of the two electrodes. The seed time series were then defined as the weighted average of the time series within the seed region, with the normalized electric field strength as the weighting factor (Reg_Emax_).

#### 2.3.3 Whole-brain functional connectivity – seed to voxel analyses

Rs-fMRI data were collected before, during, and after tDCS. The data collected during tDCS were divided into three blocks of 170 volumes (duration 7 min and 5 seconds per block) leading to five rs-fMRI blocks of 170 volumes: pre, during-vol1-170, during-vol171-340, during-vol341-510, and post. For all five blocks, functional connectivity profiles were computed using three different seed regions (anode, cathode, E_max_). Baseline (pre) rs-fMRI data were compared with data during and post-stimulation to investigate the effects of sham and active tDCS on functional connectivity. For all three seed regions, the following first-level analyses were computed to examine the main effects of time (pre versus during/post tDCS): during/post_sham_ < pre_sham_, during/post_sham_ > pre_sham_, during/post_active_ < pre_active_, during/post_active_ > pre_active_. This led to 24 (2 conditions: sham and active, 4 comparisons: pre vs during-vol1-170, pre vs during-vol171-340, pre vs during-vol341-510, and pre vs post, and 3 seed regions) first-level analyses per subject. In addition, baseline differences in functional connectivity were investigated by examining the effect of condition (sham versus active) (pre_sham_ > pre_active_, pre_sham_ < pre_active_). Subsequently, second-level analyses were performed to investigate if there are significant effects of tDCS on functional connectivity on the group level. Voxels were considered significant at a level of p < 0.001. Cluster extend thresholds were then computed to include only significant clusters at the p < 0.05 level (FWE correction).

#### 2.3.4 Dynamic effects on network connectivity

As tDCS is increasingly viewed as a tool to modulate brain networks, it was hypothesized that the effects of tDCS would propagate through functional brain networks. Hence, in addition to the whole brain seed-to-voxel analyses, it was also investigated how tDCS affects functional connectivity within resting-state networks. Templates for these resting-state networks from previous work by Yeo et al. (2011) and Buckner et al. (2013) were used. These templates were merged to also include the cerebellum in the analysis.

Firstly, for each subject and for every network, the network engagement was defined as the total electric field strength within that network, divided by the total electric field strength (times 100 to express this in percentage). Overall network engagement was subsequently defined as the average network engagement over all subjects.

Secondly, the degree was used to quantify the functional connectivity within the resting-state networks that are highly engaged using this tDCS montage. The degree of an individual node, in this case the three seed regions (anode, cathode, E_max_), equals the sum of the functional connectivity strengths between that region and all other voxels within the network (Rubinov & Sporns, 2010). Connectivity between the seed regions and the rest of the brain voxels was computed using first-level analyses for every individual, every time-window, and every seed region. In this case, the undirected weighted degree was computed for the rs-fMRI data collected during sham and active tDCS:

$$Deg_{s}(t)=\sum_{i\in N}FC_{si}(t)$$

With s representing the seed region, i the voxels within the network, and $FC_{si}$ the functional connectivity between seed region s and voxel i. N are all the voxels within the network, and t is the time-window. Interpretation of negative functional connections is ambiguous, hence we focused here on the subset of positive $FC_{si}$ values.

Paired-sample t-tests were performed to compare the functional connectivity within the resting-state networks before and during/after tDCS.

## 3 Results

For the final analyses, data of 41 subjects were used (mean age = 22.78 years old, standard deviation = 2.58 years). Four subjects had missing or incomplete rs-fMRI data and two subjects displayed excessive motion during MRI scanning, defined as mean framewise displacement >0.3 mm.

### 3.1 Insight into tDCS-induced electric fields, seed regions, and network engagement

**Fig. 2.**
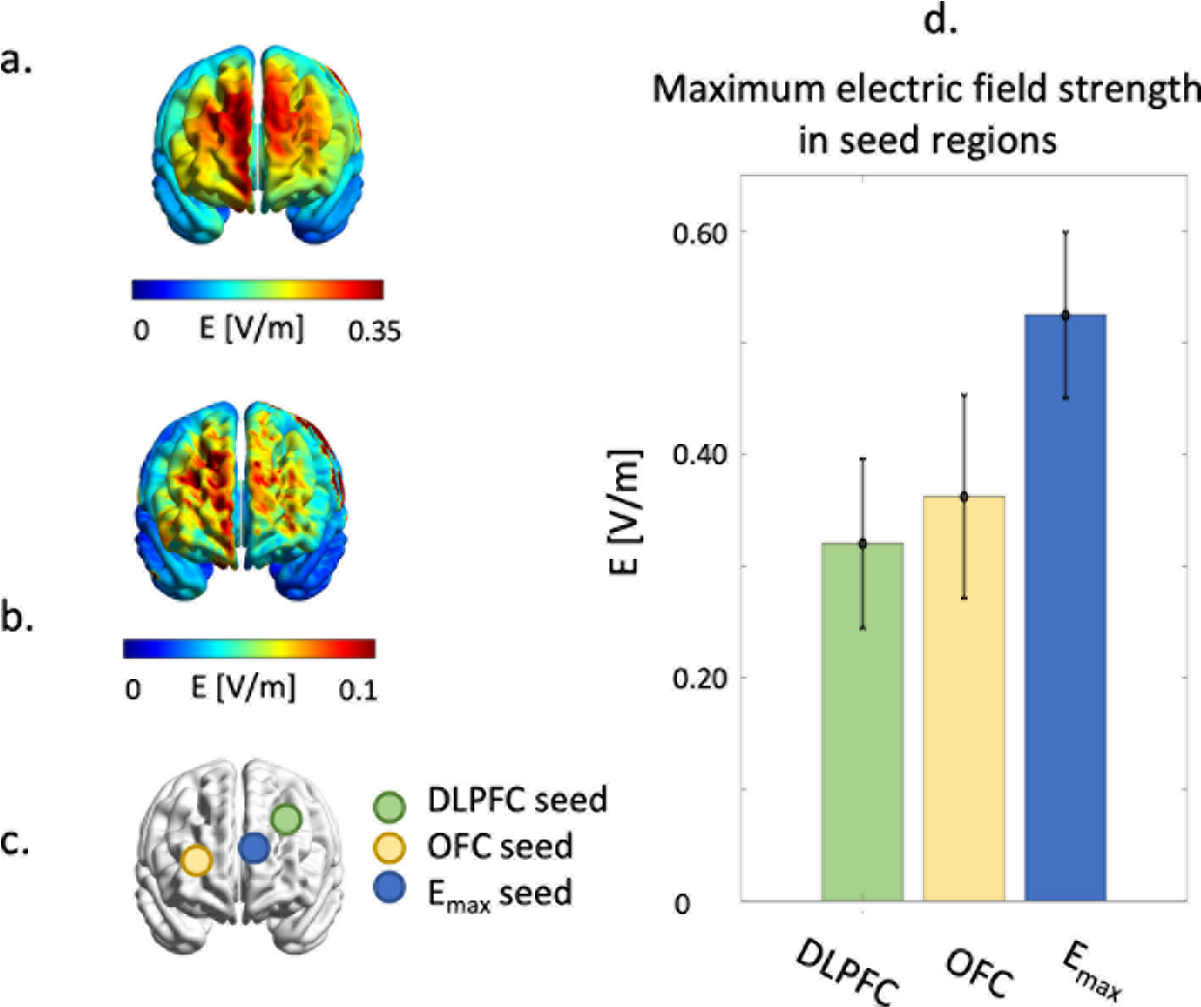
Average and standard deviation of tDCS-induced electric field distribution over all subjects with the anode placed at the left DLPFC and the cathode over the right OFC (a, b). (c) Shows the average location of the seed regions. The subject-specific locations of the DLPFC and the OFC were saved in the neuronavigation system. Subsequently, the location of the E_max_ seed is derived from the individual electric field simulations. In (d), the variability in maximum electric field strengths in the seed regions is shown.

tDCS-induced electric field simulations were used in the derivation of the seed time-series. For all three seed regions, the electric field strengths were used as weighting factors for the time-series within that region. The E_max_ seed region was located at the position where the field strength was highest. Figure 2a and 2b show the average and standard deviation of the tDCS-induced electric fields over all subjects. As can be seen, the tDCS-induced electric field distribution mostly encompasses the frontal brain regions. Figure 2c shows the locations of the seeds (averaged over all subjects). As is also shown in Figure 2a, the region where the electric field strength is highest is in the middle between the tDCS anode and cathode. The variability of the maximum electric field strengths within the seed regions is shown in Figure 2d. A detailed overview about individual electric field distributions and locations of seed regions can be found in Supplementary Material D.

**Fig. 3.**
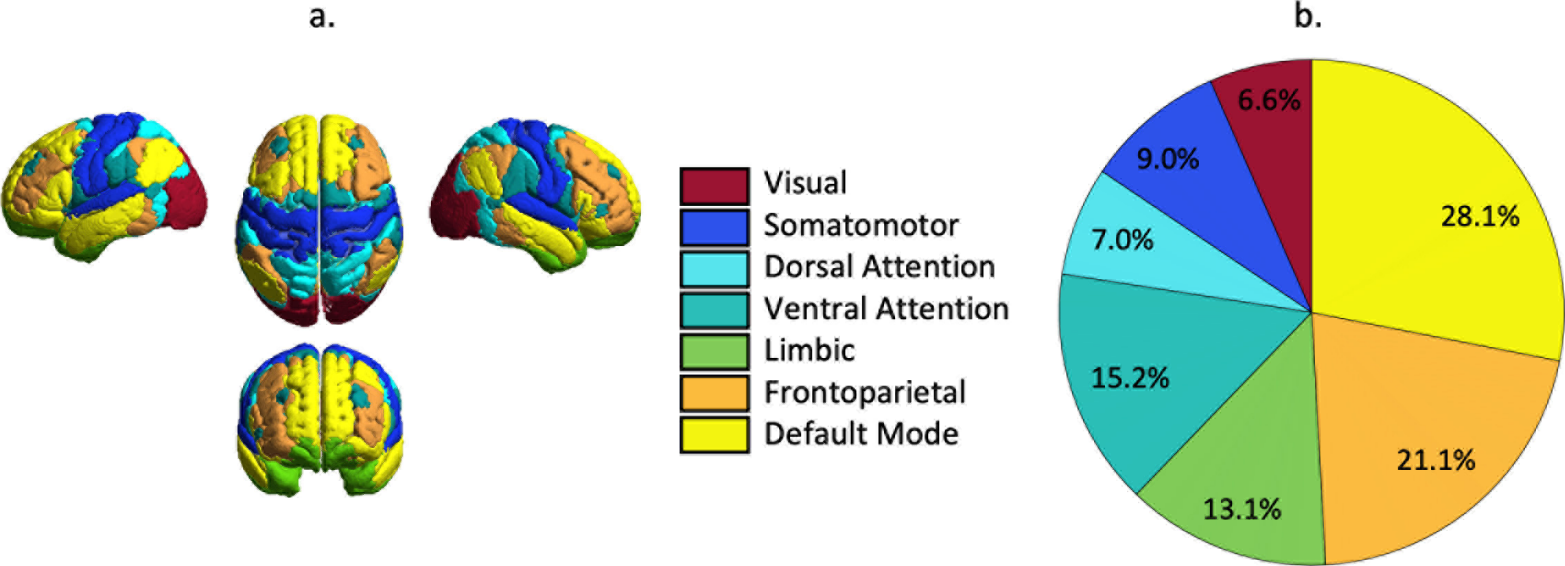
Network engagement. (a) shows the template of the resting-state networks from previous work from Yeo et al. and Buckner et al. The network engagement ( b) was computed by combining the information about the tDCS-induced electric field distribution and resting-state network patterns. The pie chart represents the averaged network engagement over all subjects.

Figure 3a shows the distribution of the resting-state networks defined by Yeo et al. (2011) and Buckner et al. (2013) and in Figure 3b, the network engagement averaged over all subjects is shown. Consistent with what was expected based on the tDCS electrode positions, the default mode network (DMN) and the frontoparietal (FP) network are predominantly being stimulated (network engagement of 28 and 21% respectively). In addition, the ventral attention (VAN, 15%) and the limbic networks (13%) are also involved in the stimulation. In line with the assumption that tDCS effects propagate throughout the brain in functional networks, it is expected that the effects on functional connectivity will be found in one of these four networks showing high engagement.

### 3.2 Whole-brain functional connectivity changes

The effects of both sham and active tDCS on seed-to-voxel connectivity were computed on the group level, using three seed regions and 4-time windows: pre versus 3-time windows during tDCS and one window after tDCS. The regressors were computed by weighting the time series within the seed region according to the strength of the tDCS-induced electric field. Importantly, we also generated unweighted regressors and observed that the weighting had minimal impact on the resulting regressor (see Supplementary Material C).

**Fig. 4.**
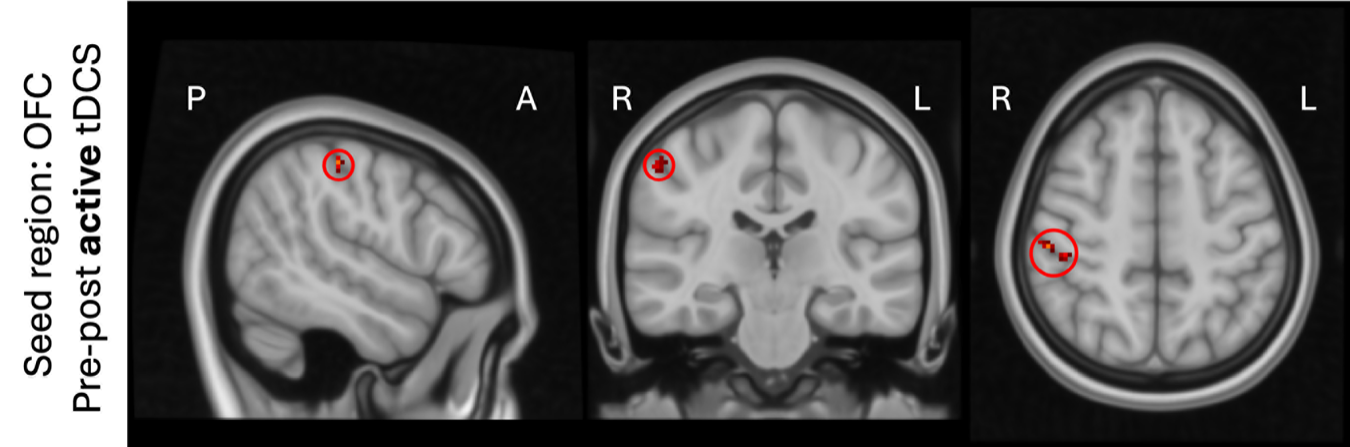
Cluster that shows significant effects of tDCS on functional connectivity when comparing rs-fMRI data before and after active tDCS using the position under the OFC electrode as seed region for connectivity analysis (highlighted with red circles to emphasize the location).

No findings of changes in functional connectivity during active tDCS were reported. Though, after active tDCS functional connectivity changed when the OFC was used as seed region. Connectivity between this seed and a cluster within the right postcentral gyrus was increased (see Fig. 4, Table 1).

**Table 1.** Statistical details about the clusters showing effects on functional connectivity.

						MNI		
Seed region	Comparison	Figure	Brain region	p_FWE-corr_	Cluster size	X	Y	Z
OFC	Pre-post active tDCS	5	Postcentral gyrus (R)	0.01	86	42	-30	54
OFC	Pre-during (TW2) sham tDCS	6a	Precuneus (bilateral) Midline (in between gray matter)	<0.01 0.03	500 67	4 0	-56 42	26 -12
DLPFC	Pre-post sham tDCS	6b	Temporal superior lateral gyrus (L)	0.05	60	-46	10	26
E_max_	Pre_sham -_ Pre_active_	6c	Parietal inferior angular gyrus (R)	0.004	98	48	-50	32

**Fig. 5.**
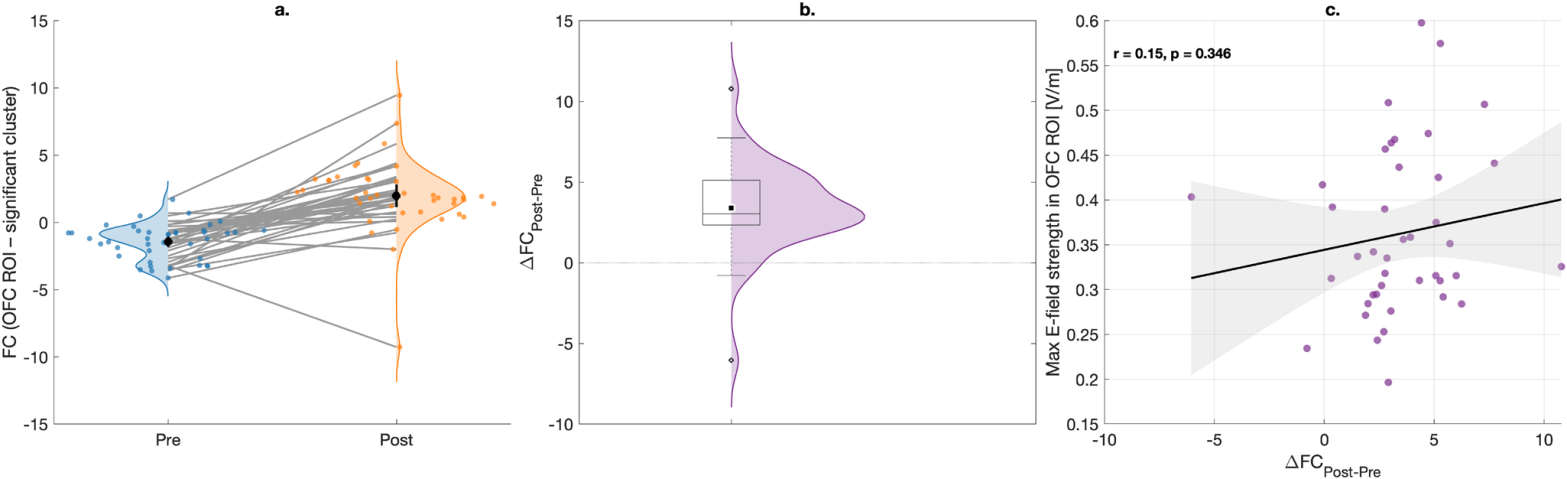
Results of post-hoc analyses confirmed that the functional connectivity increased between the OFC and a region within the right postcentral gyrus (a, b). In (c), the changes in functional connectivity are shown versus the maximum electric field strength in the seed region.

Post-hoc analysis confirmed that the connectivity between the OFC seed region and the significant cluster increased in most subjects (Fig. 5a, b). These changes in functional connectivity were not related to the maximum electric field strength in the OFC seed region (r = 0.15, p = 0.35, Fig. 5c).

**Fig. 6.**
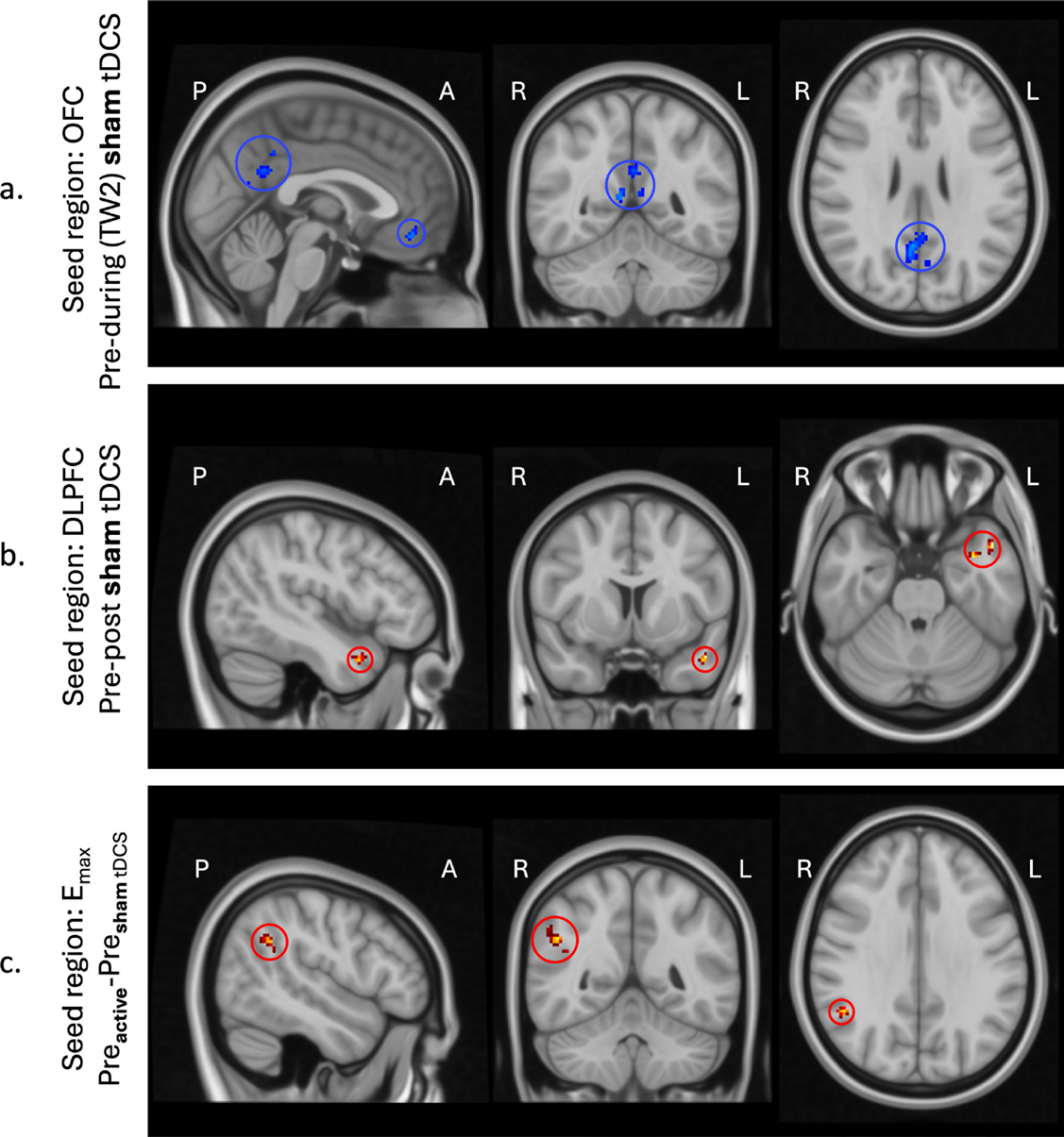
Overview of the significant clusters after sham tDCD (a and b) and when comparing baseline scans (c). Regions exhibiting increased functional connectivity are highlighted with red circles, while regions showing decreased functional connectivity are indicated with blue circles to emphasize their locations.

Sham stimulation also affected functional connectivity. During sham tDCS in the second time-window (TW2), decreased functional connectivity was found between the OFC and two clusters; one in the precuneus (bilateral) end one smaller cluster on the midline in the frontal part of the brain. Additionally, after sham tDCS, functional connectivity between the DLPFC and the temporal superior lateral gyrus was increased (Fig. 6b).

Moreover, a significant cluster was found when comparing differences in baseline scans using the Efield-derived seed region. Functional connectivity between the E_max_ seed region and the inferior angular gyrus was higher for subjects receiving active tDCS compared to sham tDCS (see Fig. 6c). As no changes in functional connectivity were observed during or after stimulation using the E_max_ seed region, these results were not considered further. Detailed info about these significant clusters can be found in Table 1.

### 3.3 Network effects

**Fig. 7.**
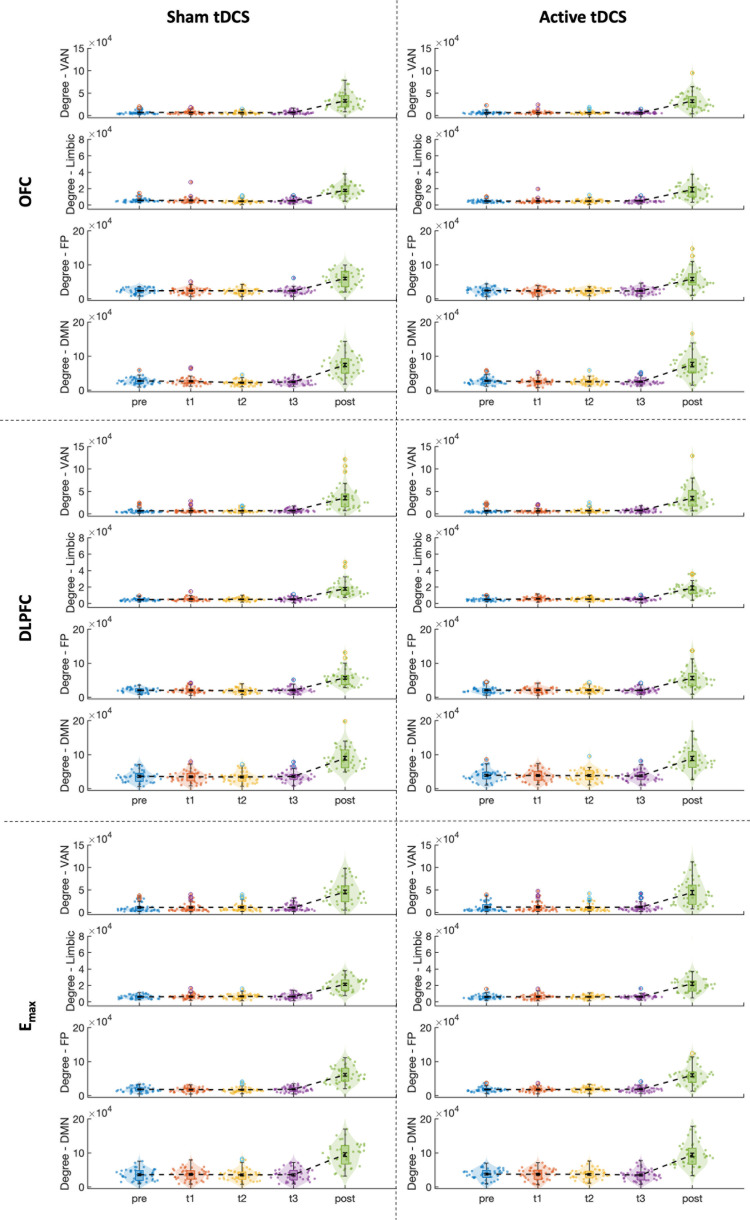
Visual representation of the network connectivity, quantified using the degree. DMN = default mode network, FP = frontoparietal, VAN = ventral attention network.

The effects of sham and active tDCS on functional connectivity were also investigated on the network level, specifically by computing the degree between the seed region and the networks that were mostly engaged in the stimulation (default mode network, frontoparietal network, ventral attention network, and limbic network, see Fig. 3) for every time window. Results can be found in Figure 7, with statistical details in Table 2.

**Table 2.** Overview of p-values when comparing network connectivity, quantified using the degree, before and during/after sham and active tDCS (*p < 0.05*, **p < 0.05/96).

		Sham tDCS				Active tDCS			
		TW1	TW2	TW3	post	TW1	TW2	TW3	post
OFC	DMN	0.46	<0.01*	0.05*	<0.01**	0.15	0.19	0.11	<0.01**
	FP	0.94	0.47	0.74	<0.01**	0.16	0.28	0.43	<0.01**
	VAN	0.80	0.43	0.93	<0.01**	0.65	0.58	0.92	<0.01**
	Limbic	0.81	0.01*	0.11	<0.01**	0.92	0.92	0.46	<0.01**
DLPFC	DMN	0.37	0.16	0.82	<0.01**	0.70	0.67	0.36	<0.01**
	FP	0.90	0.05*	0.87	<0.01**	0.97	0.74	0.59	<0.01**
	VAN	0.73	0.72	0.45	<0.01**	0.36	0.58	0.43	<0.01**
	Limbic	0.04*	0.16	0.08	<0.01**	<0.01*	0.27	0.57	<0.01**
E_max_	DMN	0.66	0.80	0.97	<0.01**	0.89	0.75	0.22	<0.01**
	FP	0.24	0.29	0.71	<0.01**	0.82	0.62	0.67	<0.01**
	VAN	0.57	0.79	0.55	<0.01**	0.99	0.46	0.75	<0.01**
	Limbic	0.38	0.189	0.32	<0.01**	0.65	0.99	0.66	<0.01**

During both sham and active tDCS, some significant effects on network connectivity were found. During sham stimulation, functional connectivity changed between the OFC and the DMN in time-windows 2 and 3, between OFC and limbic network in time-window 2, between the DLPFC and the frontoparietal network in time-window 2, and between the DLPFC and the limbic network in time-window 1 (*p < 0.05). During active stimulation, the connectivity changed between the DLPFC and the limbic network in time-window 1 (*p < 0.05). After tDCS, the degree significantly increased in almost all cases. Whereas the effects during stimulation did not survive multiple comparison correction (Bonferroni correction for multiple comparisons **p < 0.05/96), these effects after tDCS remained significant after Bonferroni correction. Since these effects were observed following both sham and active tDCS, they cannot be directly attributed to the tDCS itself.

## 4 Discussion

In our research, we took advantage of the unique benefits of combining tDCS with resting-state functional imaging to investigate the effects of tDCS on functional brain connectivity over time. To get more insights into the mechanisms of tDCS, we increased the precision and personalization of our analysis by using individual simulations of tDCS-induced electric fields. Contrary to our expectations, the results did not provide evidence in support of our hypotheses. Specifically, the sliding-window analysis did not show progressively increasing tDCS effects on functional connectivity over time for any of the seed regions (anode, cathode, or E_max_). Moreover, we found no reliable indication that tDCS-induced connectivity changes originated preferentially from regions experiencing the strongest electric fields, nor that active stimulation consistently modulated brain regions or distributed network connectivity relative to sham.

### 4.1 After-effects of tDCS on functional connectivity

#### 4.1.1 Whole brain effects

When comparing rs-fMRI data pre- and post-active tDCS, functional connectivity increased between the OFC seed region and the right postcentral gyrus. Although prior studies have demonstrated that tDCS effects can propagate across widespread brain networks, none have specifically reported functional connectivity changes in the postcentral gyrus, particularly when using a montage comparable to that employed in the present study.

No effects of active tDCS on functional connectivity were found when the location of the anode (DLPFC) was taken as a seed region. In contrast, Park et al. (2013) reported increased connectivity between the DLPFC and the right hemisphere and decreased connectivity with the regions around the left stimulation site after active tDCS compared to sham, even though the stimulation intensity was lower (1 mA instead of 1.5 mA). However, this study was a between-subject design and used a lower threshold for statistical significance (cluster threshold 17 voxels).

Also, no effects on functional connectivity were found when the E_max_ seed region was used. So even though we hypothesized that the effects of tDCS might be caused by locations that receive high electric field strengths, this was not confirmed in this study. The location of the E_max_ seed region differs between subjects due to differences in tDCS-induced electric field distributions. These differences are inherent consequences of the subjects’ individual brain characteristics, such as the shape and thickness of the skull and the exact individual gyral folding pattern, and have been extensively discussed in previous literature (Laakso et al., 2016; Mikkonen et al., 2020). Differences in electric field distributions—and the resulting variability in the location of the E_max_ seed region—may have led the seed to fall within slightly different functional regions or networks across individuals, potentially producing heterogeneous effects that are not detectable at the group level. Future research employing personalized electrode placements to optimize electric field strength within a predefined target region is needed to more definitively determine whether tDCS exerts its effects via regions experiencing the strongest induced electric fields. A comparable targeting approach based on computational modeling has previously been proposed by us for optimizing TMS coil positioning (Klooster et al., 2022).

A post-hoc analysis showed that the changes in functional connectivity between the OFC and the postcentral gyrus were not related to the strength of the electric field within the seed region. Importantly, the relation between tDCS intensity—and consequently the induced electric field strength—and response is not fully understood (Esmaeilpour et al., 2018). The importance of the induced electric field strength was previously underlined by a cadaver study (Vöröslakos et al., 2018), which showed that only 25% of the currents applied to the scalp reach the brain. This suggests that very high intensities are required to induce significant effects on neuronal activity. Recent work shed more light on the concrete intensities required to cause neuronal activity and showed that electric fields of 0.35 V/m can modulate firing rate in hippocampal neurons in rats (Farahani et al., 2024). Application of a standard stimulation intensity (such as 1.5 mA in this study) leads to variability in the maximum tDCS-induced electric field strengths (Evans et al., 2020; Laakso et al., 2019). This variability, quantified by the maximum strength within the seed regions, was also observed in our study (Fig. 2d). So, in some of our subjects, the electric field strength induced by tDCS may not have been high enough to alter membrane potentials.

It remains to be determined how associations between electric field strength and functional connectivity translate to relationships with clinical metrics. Although we did not observe a link between field strength and changes in functional connectivity in this study, prior work has reported associations between electric field strength and clinical response. Wischnewski et al. used an alternative novel meta-analytical approach to investigate the relation between tDCS-induced electric field strengths and the effects of stimulation on working memory (Wischnewski et al., 2021, 2024). Electric field strength in different brain regions was shown to be correlated with various aspects of working memory. For example, stronger electric fields in the inferior frontal regions were related to enhanced working memory accuracy (Wischnewski et al., 2024).

Interestingly, we also observed changes in functional connectivity following sham stimulation. Although sham tDCS is intended to mimic the sensations of active stimulation without delivering a sustained current, research has shown that sham protocols can themselves be a source of variability and may produce biological effects beyond transient sensations, potentially confounding comparisons between active and sham conditions (Fonteneau et al., 2019; Palm et al., 2013).

#### 4.1.2 Network effects

On the network level, the degree increased in all networks that are engaged in this electrode montage; the frontoparietal, default mode, ventral attention, and the limbic network using all three seed regions after but not during stimulation. This means that the connectivity between the seed region and all other voxels within the network increased after tDCS. These effects were similar after active and sham tDCS. Hence, in contrast to our hypothesis, no after-effects on network level were found that could be related to active tDCS.

The increase in functional connectivity, reflected by the degree, is observed after both active and sham stimulation and therefore unlikely to reflect a direct effect of active stimulation. Rather, it may be related to time-dependent changes occurring over the course of the experimental session. Notably, stimulation ended after time-window 4 so data during time-window 5 was collected without stimulation. The cessation of stimulation may have induced changes in participants’ brain state, possibly reflecting a shift from stimulation-related arousal to a more relaxed or resting state. This state during time-window 5 may reflect a greater level of relaxation than during the baseline measurement (time-window 1), given that the experimental session was approaching its end. Because the sham protocol included ramp-up and ramp-down phases to mimic the sensations of active stimulation as closely as possible, similar effects may have occurred following both active and sham tDCS.

Other studies did show network-level effects of tDCS on functional connectivity (Keeser et al., 2011; Peña-Gómez et al., 2012). Both studies showed an effect within the DMN. In both cases, the DMN was derived using independent component analysis and the connectivity analyses was performed using the whole network as seed region. This is in contrast with our work, in which we used small seed regions and computed the connectivity between the seed and the network. Additionally, both studies stimulated at 2 mA and had a relatively low number of included subjects (13 and 10 in Keeser et al. and Peña-Gómez et al. respectively).

#### 4.1.3 No effects during active stimulation

Even though it was hypothesized that the effects of tDCS on functional connectivity would build up and intensify during the stimulation period, this study did not confirm this. No effects of active tDCS on functional connectivity were found using any of the seed regions, in any of the time-windows during stimulation. Three earlier studies (Bouchard et al., 2023; Leaver et al., 2022; Mondino et al., 2020) that analyzed rs-fMRI data collected during tDCS, using comparable methodology as in this work, all reported effects on functional connectivity. All studies used whole brain seed-to-voxel connectivity with the seed regions placed under the stimulation electrodes. However, Mondino et al. (2020) and Bouchard et al. (2023) both stimulated at 1 mA but for an extended duration of 30 minutes and Leaver et al. (2022) stimulated at 2 mA for only 5 minutes. As stated before, the interplay of effects between stimulation duration and intensity on the effects of functional connectivity is not well understood. Additionally, the placement of the reference electrode also differed between these studies and our work, which could also at least partly explain the different findings (Wörsching et al., 2018).

Interestingly, Mondino et al. (2020) separately analyzed different time-windows of the resting-state data, in line with our work. Effects of tDCS on functional connectivity were already found within the first 15 minutes, did not change in the second 15 minutes, and persisted after stimulation.

### 4.2 Methodological considerations

A sliding-window approach was used to compute the functional connectivity over time. The choice of the window length was set to resemble the length of our pre- and post-tDCS scans. On the one hand, shorter time windows can capture the effects of tDCS on functional connectivity in a higher temporal resolution. On the other hand, longer windows are more robust to spurious fluctuations caused by noise (Hutchison et al., 2013). Given the anticipation of gradual, intensifying effects of tDCS on functional connectivity (rather than rapid fluctuations in tDCS effects), relatively long windows were chosen. Still, no study has convincingly identified the optimal window length for this type of functional connectivity analyses (Shakil et al., 2016).

Of course, we can only interpret our findings within the realm of young non-depressed females. We cannot simply extrapolate to healthy young males, given that the stimulation responses could be sex specific on task accuracy (Dedoncker et al., 2016) or on motor performance (Frankel et al., 2025). We cannot extrapolate to older ages as with advanced age the behavioral tDCS response variability increases (Fromm et al., 2025).

## 5 Conclusion

This study investigated the effects of tDCS on functional connectivity during and after the stimulation. In addition to seed regions under the anode and cathode, a third seed region was derived from individual tDCS-induced electric field simulations. No effects of active tDCS on functional connectivity were detected during stimulation. However, following active stimulation, increased connectivity was observed between the OFC seed region and the postcentral gyrus. Taken together, the results did not support our a priori hypotheses, as active tDCS did not robustly modulate brain regions or distributed networks relative to sham, nor did we observe progressively increasing effects on functional connectivity over time for any of the seed regions (anode, cathode, or E_max_). Furthermore, there was no consistent evidence that connectivity changes preferentially originated from regions experiencing the strongest electric fields. Future research should explore the effect of tDCS on functional connectivity further and investigate the potential relation with clinical outcome measures.

## Supplementary Material

Supplementary Material

## Data Availability

Data are available for sharing upon reasonable request. The corresponding author can be contacted.
