# Supplementary material for: Simultaneous tDCS-fMRI reveals limited and inconsistent changes in functional connectivity: Insights from a temporal dynamics study
Source: Imaging Neurosci (Camb). 2026 Apr 2;4:IMAG.a.1109. doi: 10.1162/IMAG.a.1109 (PMC13047502; doi:10.1162/IMAG.a.1109)
Supplement: Supplementary Material [file IMAG.a.1109_supp.pdf]

Supplementary materials

A. Literature overview of tDCS-fMRI studies to the dorsolateral prefrontal cortex in healthy volunteers

Table S1: Effects of tDCS, applied to the dorsolateral prefrontal cortex in healthy volunteers, measured using resting-state functional connectivity, in absence of a task.

**Sequential approach: rs-fMRI – tDCS – rs-fMRI**						
Study	Electrode montage	Stimu-lation duration (min)	Stimulation intensity (mA)	N	Analysis	Outcome
Keeser et al. 2011 (Keeser et al., 2011)	Left DLPFC, right OFC	20	2	13 (men)	Network connectivity (independent component analysis)	After real tDCS, significant changes of regional brain connectivity were found for the DMN and the FPN both close to the primary stimulation site and in connected brain regions.
Peña-Gomez et al. 2012 (Peña-Gómez et al., 2012)	Left DLPFC, right OFC	20	2	10	Independent component analysis	Active tDCS showed decreased synchrony in the DMN and increased synchrony in an anti-correlated network (AN).
Park et al. 2013 (Park et al., 2013)	Left DLPFC, right OFC	20	1	25 active, 14 sham	Whole brain seed (DLPFC) to voxel analysis	Widespread changes throughout the brain: Increased rsFC between left DLPFC and frontal, temporal, and subcortical regions on the right sides. Decreased FC found between left DLPFC and frontal regions around stimulation region on the left side.
**Concurrent approach: tDCS during rs-fMRI**						
Mondino et al. 2020 (Mondino et al., 2020)	Left and right DLPFC (F3-F4)	30	1	15 (n=13 analyzed)	Left and right DLPFC as seed region (F3 and F4, MNI coordinates)	tDCS induced changes in FC between the left DLPFC and bilateral parietal regions (already within the first 15 minutes, lasting in the second 15 minutes and after stimulation).
Leaver et al. 2022 (Leaver et al., 2022)	Left DLPFC (F3) and right ventrolateral PFC (F8)	5	2	64 (n = 37 targeting DLPFC)	Node to network connectivity	tDCS increased mean FC in the OFC network and decreased connectivity between the frontoparietal network and node close to subgenual anterior cingulate cortex.
Bouchard et al. 2023 (Bouchar	Left and right DLPFC (F3-F4)	30	1	16	Left and right DLPFC as seed region (F3 and F4, MNI coordinates)	tDCS modulates FC of circuits comprising the distal (parietal, occipital, temporal) and proximal

d et al., 2023)						(frontal) regions during and after the stimulation.
Tu et al. 2021	Right DLPFC and left OFC	20	2	81	Co-activation pattern (CAP) analysis using rDLPFC and lOFC as seed regions	Anodal tDCS at the rDLPFC and cathodal tDCS significantly modulate occurance rates of CAPs, and perturb transition between CAPs and non-CAP states.

B: Variation in seed regions

Table S1 represents an overview of the MNI coordinates for the seed regions used for functional connectivity analyses. The OFC and DLPFC locations were derived from neuronavigation whereas the E_{max} location was defined as the position where the individual tDCS-induced electric field strengths were strongest. These locations are shown in Figure S1.

Table S2: MNI coordinates of the seed regions used for functional connectivity analyses.

Patient ID	OFC			DLPFC			E_{max}		
	x	y	z	x	y	z	x	y	z
1	25	66	15	-42	28	50	-12	46	44
2	16	65	7	-31	21	59	10	64	18
3	17	71	13	-37	31	55	-34	42	20
4	38	65	11	-36	47	41	14	52	32
5	25	69	8	-35	40	46	-18	58	22
6	27	67	10	-37	27	55	-26	40	42
7	28	58	29	-35	33	51	-10	44	44
8	37	64	25	-43	27	57	16	42	40
9	27	69	9	-34	25	60	-28	48	32
10	31	70	14	-35	42	47	12	66	16
11	21	68	18	-37	26	58	-20	26	58
12	13	70	4	-35	37	45	-6	54	28
13	23	63	-14	-40	27	36	12	56	34
14	31	62	21	-35	35	50	24	44	36
15	36	61	19	-40	35	47	10	52	40
16	35	66	12	-38	24	56	26	38	30
17	24	69	10	-39	32	49	-14	48	34
18	27	67	10	-37	27	55	-30	46	24
19	26	73	9	-34	36	56	10	64	20
20	27	67	10	-37	27	55	8	52	38
21	27	67	10	-37	27	55	20	60	10
22	37	59	11	-35	26	51	-8	46	44
23	27	67	10	-37	27	55	-26	46	32
24	27	67	10	-37	27	55	8	62	18
25	16	71	21	-36	35	59	-32	38	32
26	30	67	-3	-30	27	56	-6	42	48
27	32	62	15	-33	9	64	16	42	44
28	30	66	4	-30	20	65	16	28	54
29	22	69	12	-35	13	64	-18	42	34
30	11	71	13	-42	30	49	-34	48	16
31	35	64	11	-40	28	55	32	34	30
32	32	68	19	-39	25	58	16	28	52

33	31	66	12	-32	23	58	26	32	44
34	26	70	11	-41	27	55	-14	58	22
35	24	72	11	-35	21	58	20	38	48
36	36	68	10	-36	13	65	20	42	38
37	34	69	2	-35	34	50	28	40	30
38	26	64	-10	-37	29	54	18	40	40
39	27	67	10	-37	27	55	8	58	28
40	20	63	-1	-43	19	53	-38	40	24
41	30	67	-2	-38	19	55	10	64	20
**Mean**	**27**	**67**	**10**	**-37**	**28**	**54**	**0**	**47**	**33**
**std**	**7**	**3**	**8**	**3**	**8**	**6**	**21**	**10**	**12**

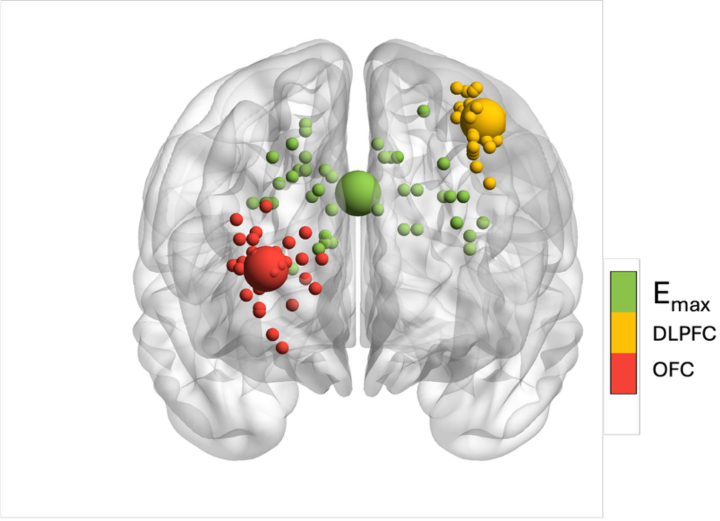

Figure S1. Visualization of the distribution of the different seed regions.

C. Weighted versus unweighted regressors

For each subject and each rs-fMRI dataset (pre-, during-, and post-tDCS), regressors were generated to perform functional connectivity analyses using three seed regions (DLPFC, OFC, and E_{max}). These regressors were computed both with and without incorporating the electric field strength as a weighting factor. The table below presents the mean and standard deviation across subjects for the correlations between the weighted and unweighted regressors. The consistently high mean values and low standard deviations indicate that applying the weighting has minimal impact on the regressors and consequently on the functional connectivity results.

Table S3: Mean and standard deviations of the correlations between regressors computed with and without incorporating the electric field strength as a weighting factor.

	PRE				During tDCS				POST			
	Session 1		Session 2		Session 1		Session 2		Session 1		Session 2	
	Mean	Std	Mean	Std	Mean	Std	Mean	Std	Mean	Std	Mean	std
DLPFC	0.999	0.001	0.999	0.001	0.999	0.001	0.999	0.002	0.999	0.002	0.999	0.001
OFC	0.998	0.003	0.999	0.002	0.999	0.002	0.998	0.003	0.999	0.001	0.999	0.001
E_{max}	0.998	0.002	0.998	0.008	0.998	0.002	0.997	0.004	0.998	0.001	0.998	0.002

D. Variation in electric field distributions

Electric field overview (1/9)

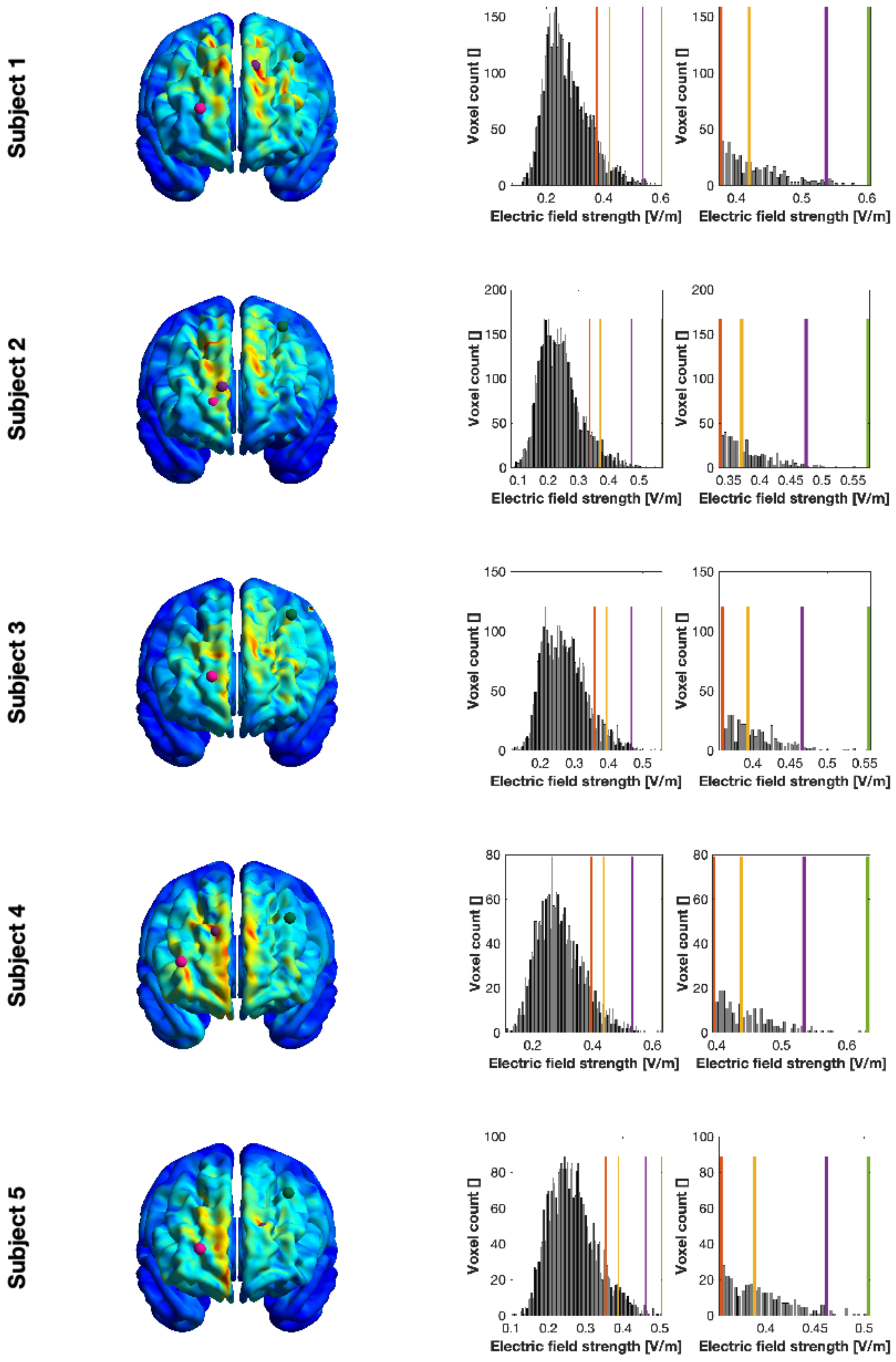

38

Electric field overview (2/9)

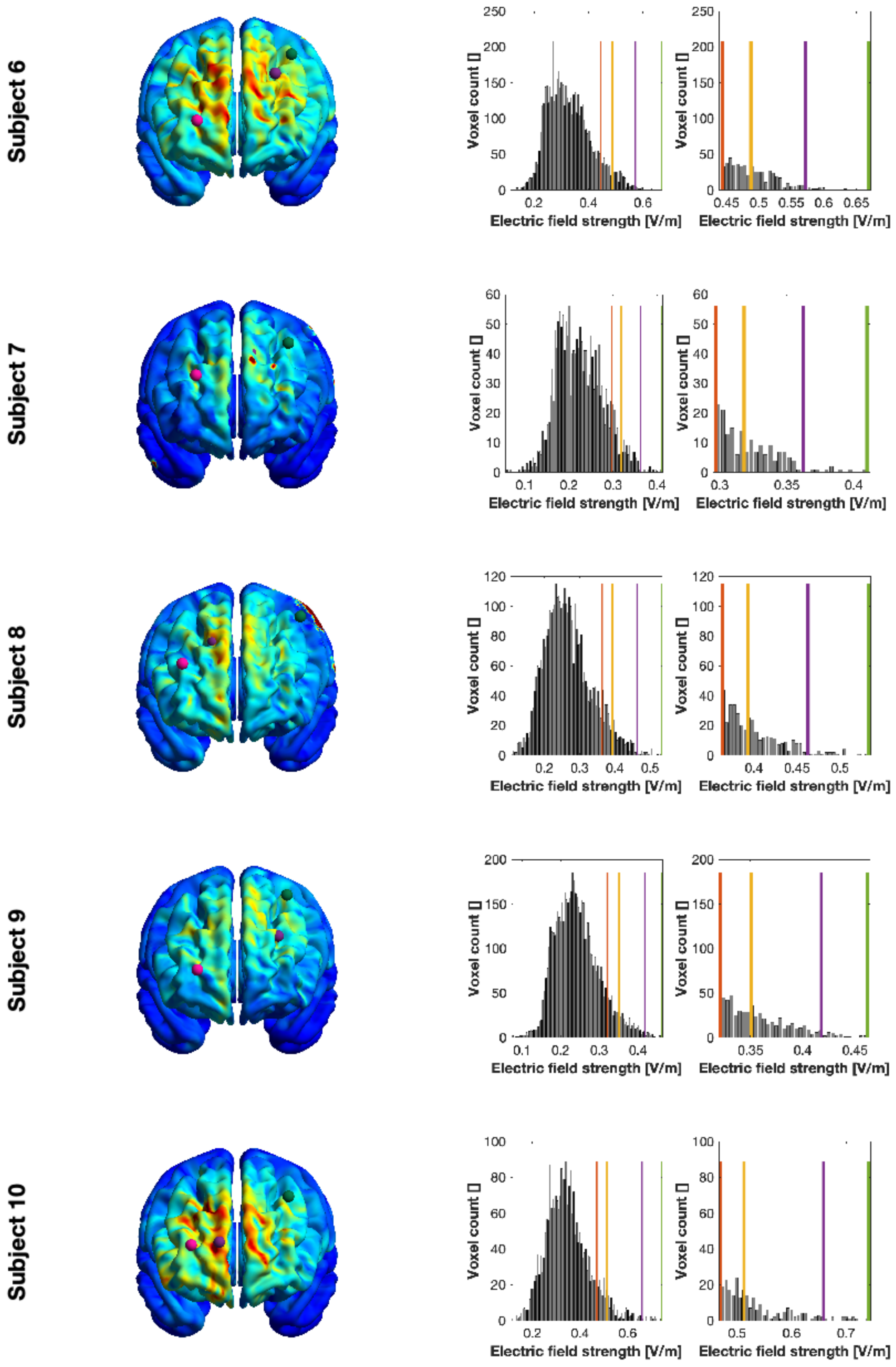

Electric field overview (3/9)

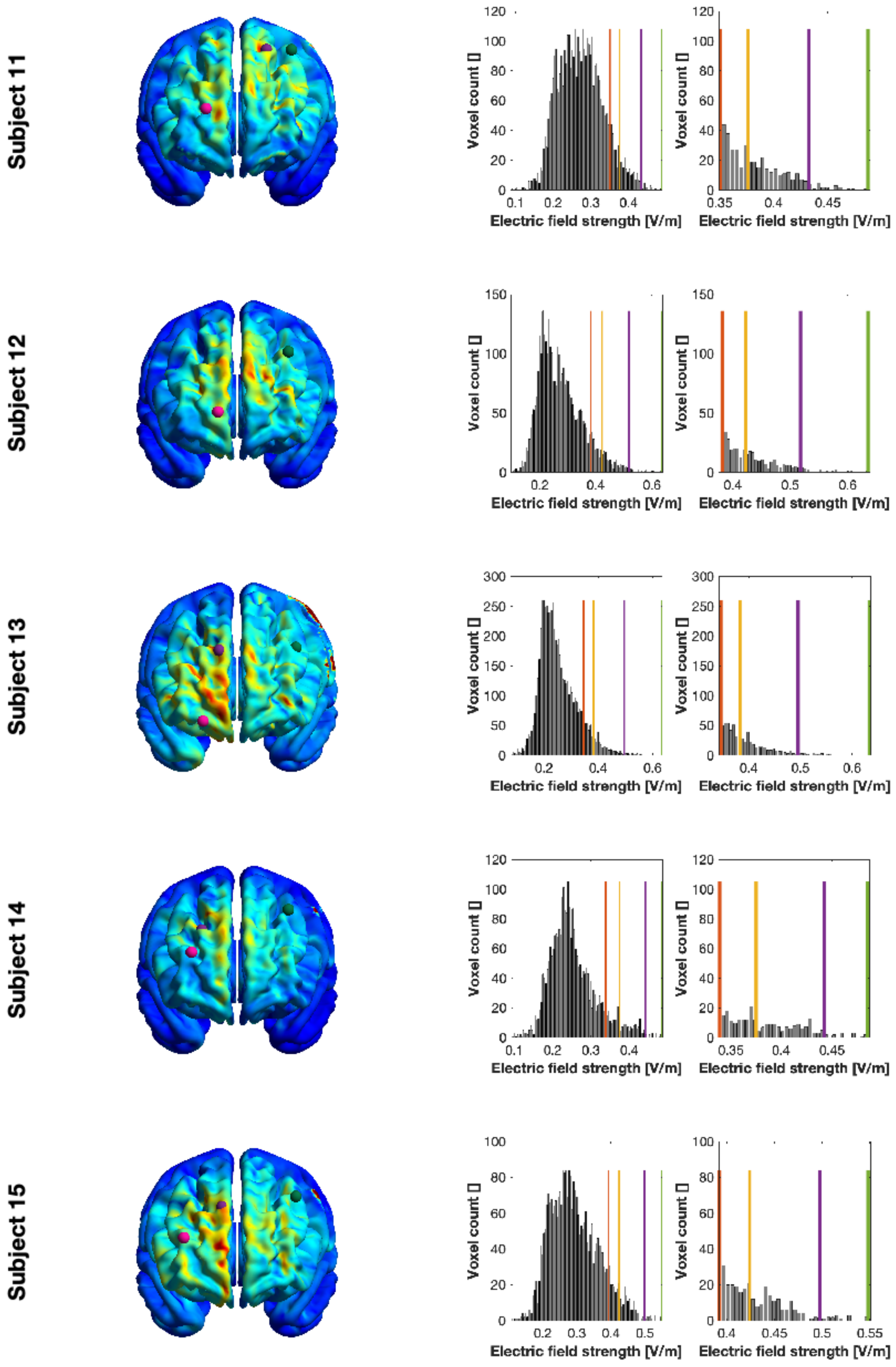

Electric field overview (4/9)

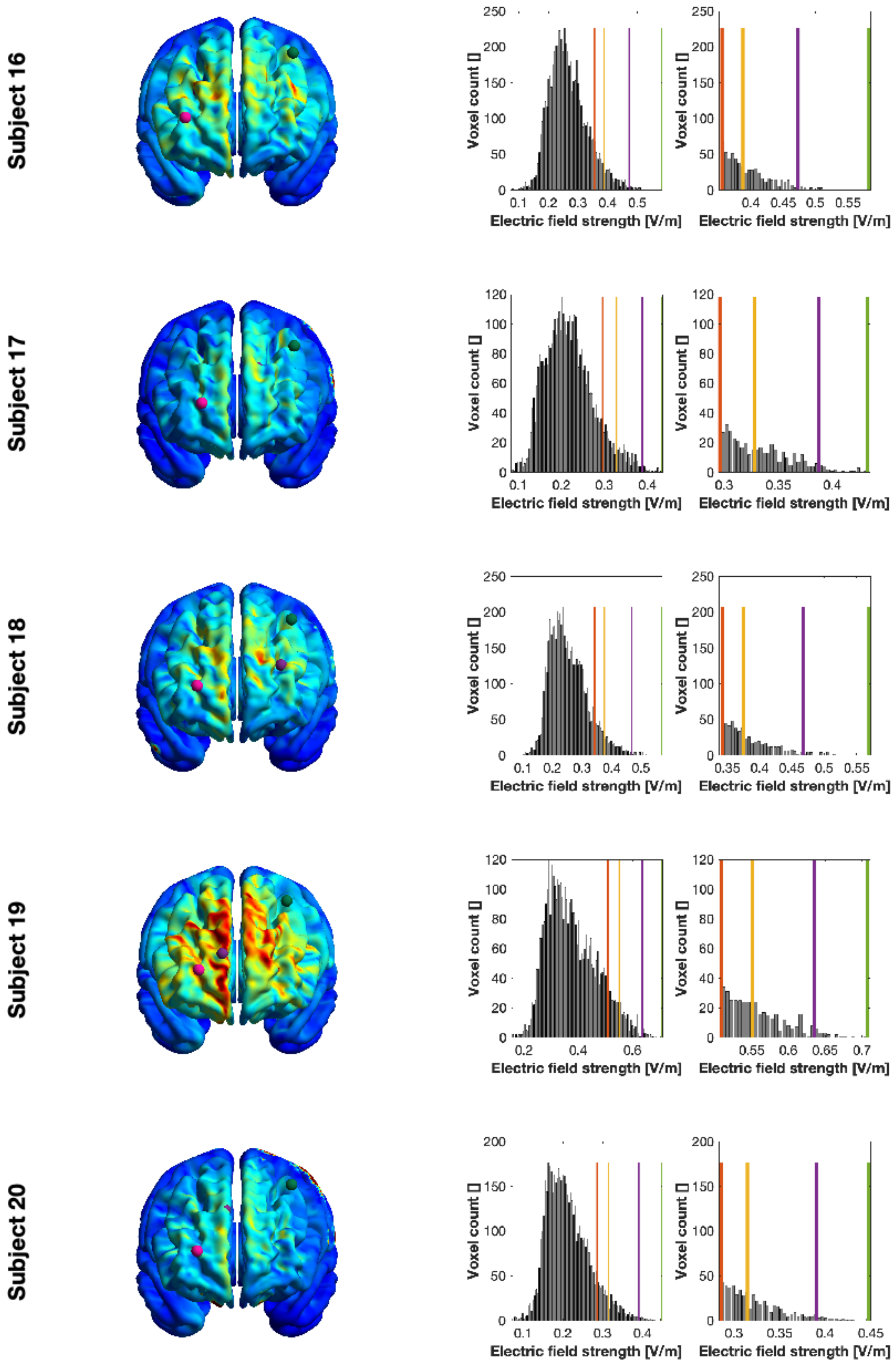

Electric field overview (5/9)

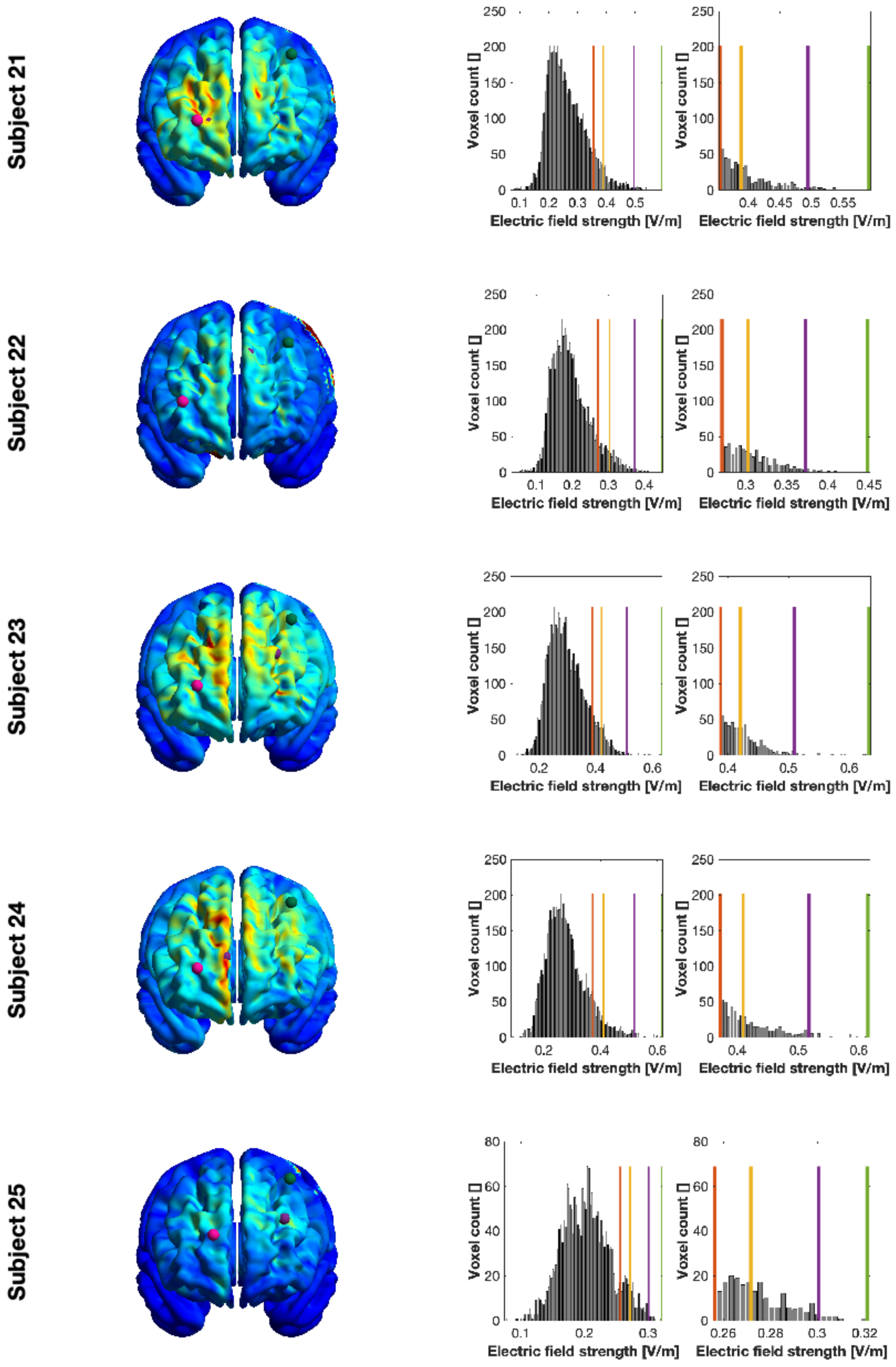

48

Electric field overview (6/9)

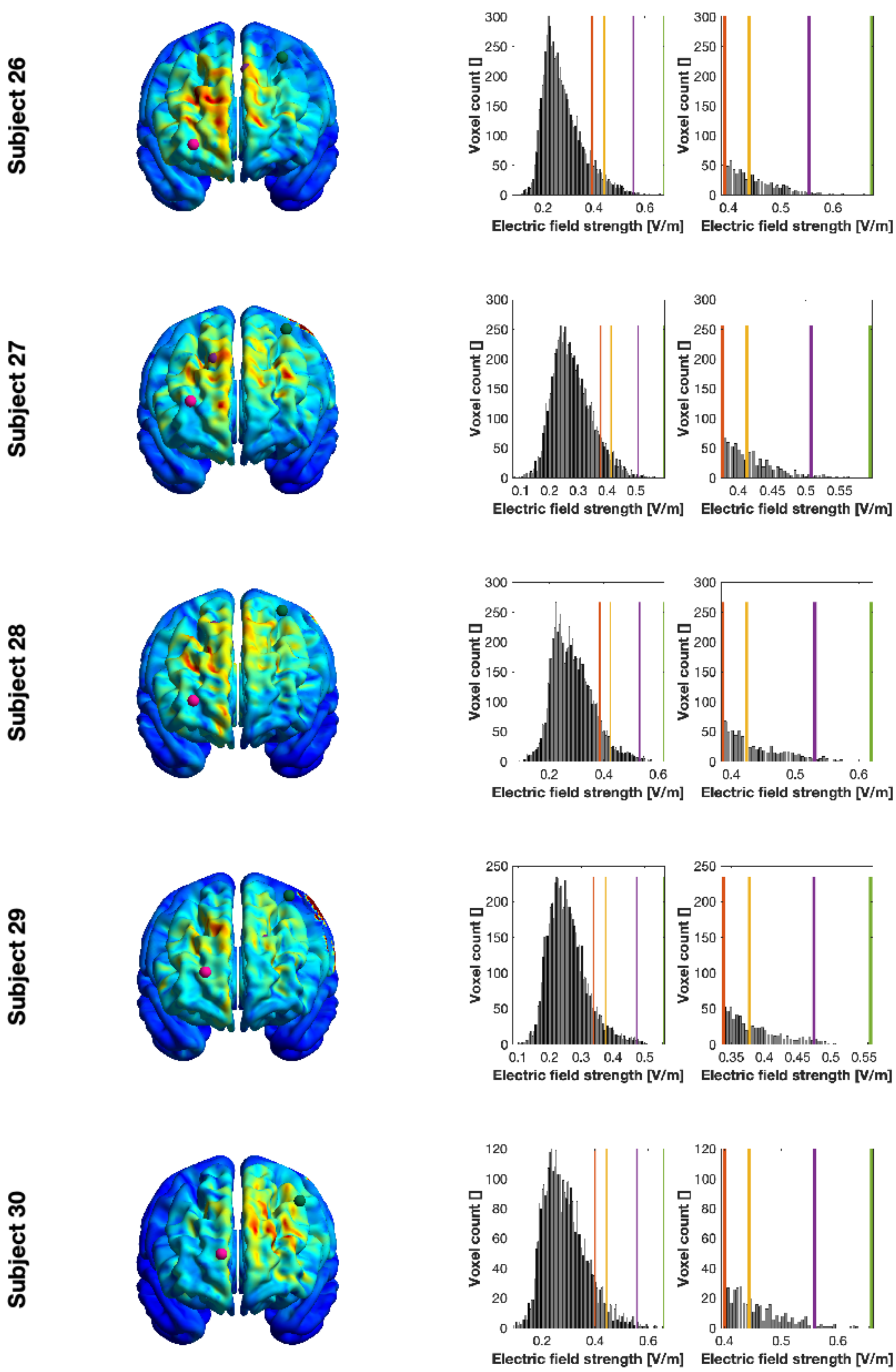

49

Electric field overview (7/9)

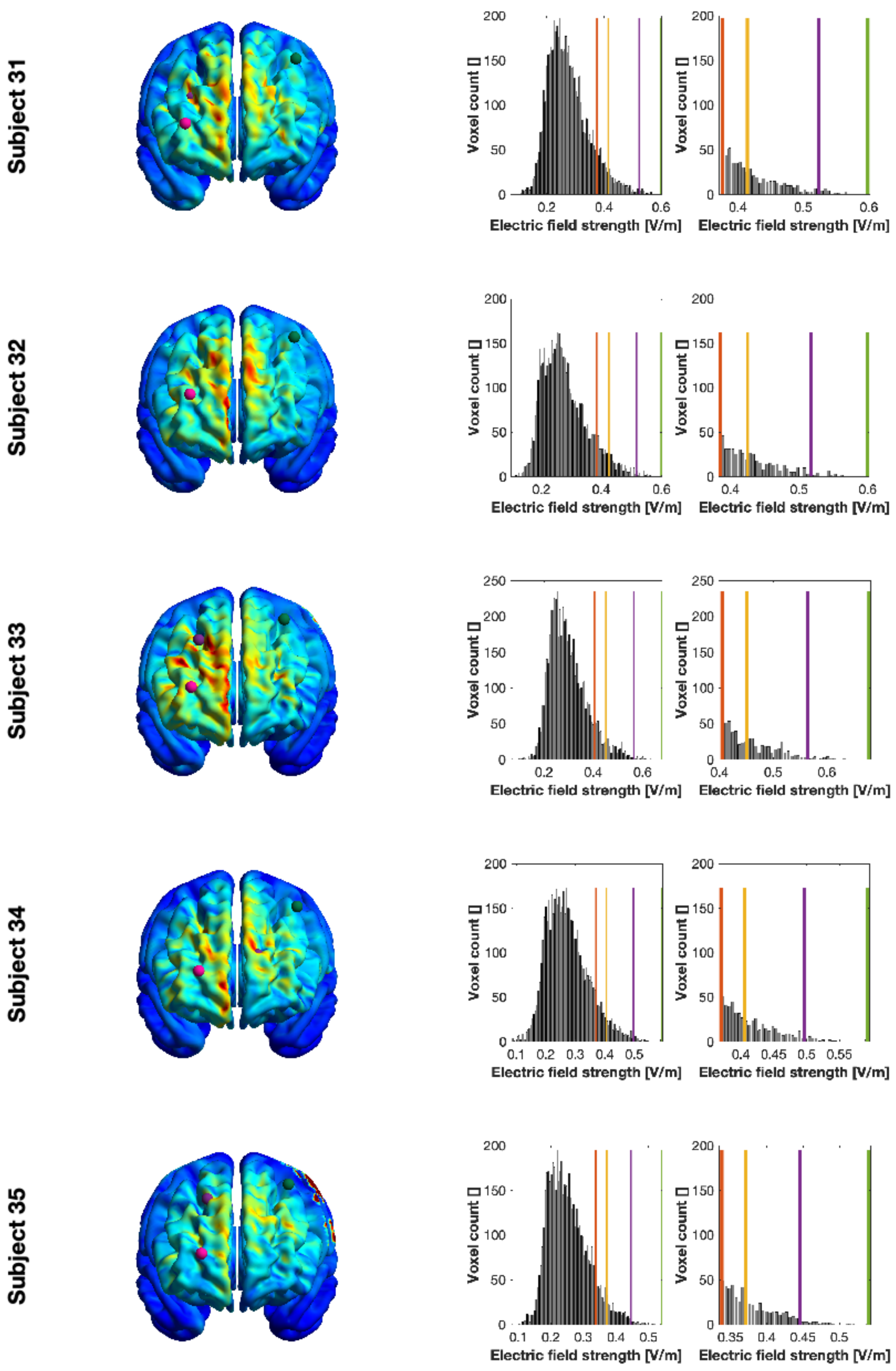

50

Electric field overview (8/9)

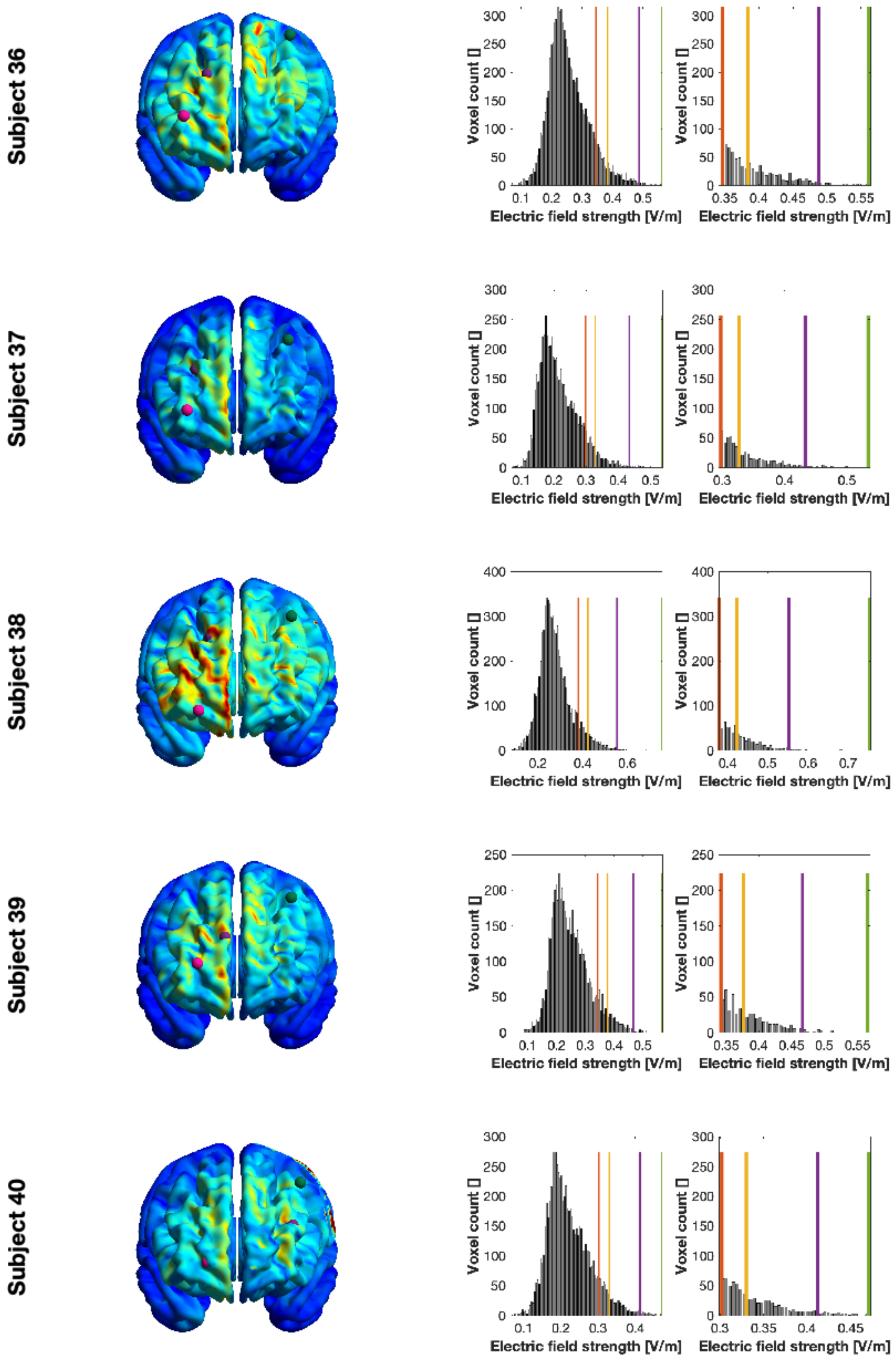

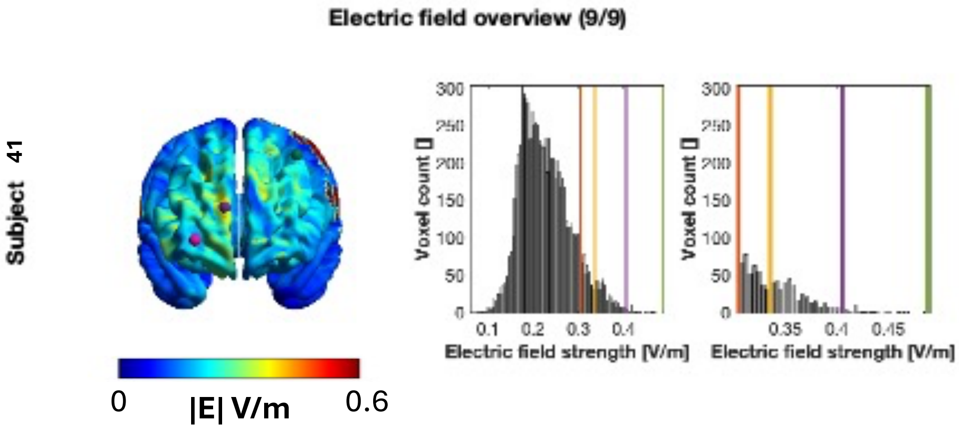

Figure S2: Overview of the individual TMS-induced electric field distributions, including visualizations of each subject's seed regions used for the functional connectivity analysis (left). The two histograms on the right display the distribution of electric field strength within the gray matter, with colored lines indicating the 90th, 95th, 99.5th, and 100th percentiles. The histograms were used to determine the location of the E_{max} seed region. A threshold of 99.5% was chosen as a compromise, representing a value sufficiently high to capture the maximum while avoiding potential outliers.
